# Tris(2,2′-bipyridine)­cobalt(II) μ_6_-oxido-dodeca-μ_2_-oxido-hexa­oxidohexa­molydate(VI)

**DOI:** 10.1107/S1600536810020775

**Published:** 2010-06-05

**Authors:** Ying Liu, Xianxi Zhang, Zechun Xue, Jian Sheng

**Affiliations:** a College of Chemistry and Chemical Engineering, Liaocheng University, Liaocheng 252059, People’s Republic of China

## Abstract

In the title compound, [Co(C_10_H_8_N_2_)_3_][Mo_6_O_19_], the Co^2+^ cation is surrounded in a distorted octa­hedral coordination by six N atoms from three 2,2′-bipyridine ligands. The distribution of Mo—O bond lengths in the Lindqvist isopolyanion is consistent with other structures containing the same unit. In the crystal, the cations and anions are linked by C—H⋯O inter­actions.

## Related literature

For general background to polyoxometalates, see: Pope & Müller (1991 ▶). For polyoxometalates modified with amines, see: Zhang, Dou *et al.* (2009 ▶); Zhang, Wei *et al.* (2009 ▶). For another structure containing the μ6-oxido-dodeca­kis­-μ2-oxido-hexaoxidohexamolydate(VI) anion see: Dahlstrom *et al.* (1982 ▶). For Co—N bond lengths in a related structure, see: Li & Xu (2009 ▶).
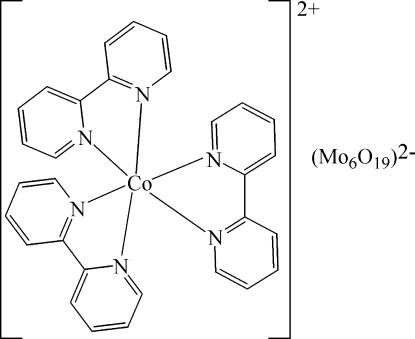


## Experimental

### 

#### Crystal data

[Co(C_10_H_8_N_2_)_3_][Mo_6_O_19_]
*M* *_r_* = 1407.12
Monoclinic, 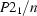

*a* = 12.310 (2) Å
*b* = 18.979 (4) Å
*c* = 17.150 (4) Å
β = 100.895 (3)°
*V* = 3934.4 (14) Å^3^
*Z* = 4
Mo *K*α radiation
μ = 2.35 mm^−1^
*T* = 296 K
0.12 × 0.10 × 0.08 mm

#### Data collection

Bruker APEXII CCD diffractometer
Absorption correction: multi-scan (*SADABS*; Bruker, 2001 ▶) *T*_min_ = 0.766, *T*_max_ = 0.834
25652 measured reflections
6500 independent reflections
4649 reflections with *I* > 2σ(*I*)
*R*_int_ = 0.041

#### Refinement

*R*[*F* ^2^ > 2σ(*F* ^2^)] = 0.032
*wR*(*F* ^2^) = 0.092
*S* = 1.00
6500 reflections
559 parameters
H-atom parameters constrained
Δρ_max_ = 0.65 e Å^−3^
Δρ_min_ = −0.54 e Å^−3^

### 

Data collection: *APEX2* (Bruker, 2004 ▶); cell refinement: *SAINT-Plus* (Bruker, 2001 ▶); data reduction: *SAINT-Plus*; program(s) used to solve structure: *SHELXS97* (Sheldrick, 2008 ▶); program(s) used to refine structure: *SHELXL97* (Sheldrick, 2008 ▶); molecular graphics: *SHELXTL* (Sheldrick, 2008 ▶); software used to prepare material for publication: *SHELXTL*.

## Supplementary Material

Crystal structure: contains datablocks global, I. DOI: 10.1107/S1600536810020775/hb5476sup1.cif

Structure factors: contains datablocks I. DOI: 10.1107/S1600536810020775/hb5476Isup2.hkl

Additional supplementary materials:  crystallographic information; 3D view; checkCIF report

## Figures and Tables

**Table 1:** Selected bond lengths (Å)

Co1—N5	2.075 (5)
Co1—N6	2.078 (5)
Co1—N1	2.079 (5)
Co1—N4	2.081 (5)
Co1—N2	2.091 (5)
Co1—N3	2.100 (5)

**Table 2:** Hydrogen-bond geometry (Å, °)

*D*—H⋯*A*	*D*—H	H⋯*A*	*D*⋯*A*	*D*—H⋯*A*
C2—H2⋯O11^i^	0.93	2.36	3.166 (9)	145
C4—H4⋯O17^ii^	0.93	2.52	3.165 (9)	127
C11—H11⋯O4^iii^	0.93	2.51	3.400 (8)	161
C12—H12⋯O2^iii^	0.93	2.47	3.277 (10)	145
C20—H20⋯O14^iv^	0.93	2.53	3.159 (9)	125
C22—H22⋯O8^v^	0.93	2.54	3.230 (9)	132
C26—H26⋯O18	0.93	2.58	3.459 (8)	157

Symmetry codes: (i) 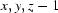; (ii) 
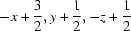
; (iii) 
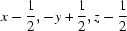
; (iv) 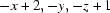; (v) 
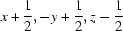
.

**Fig. 1.**
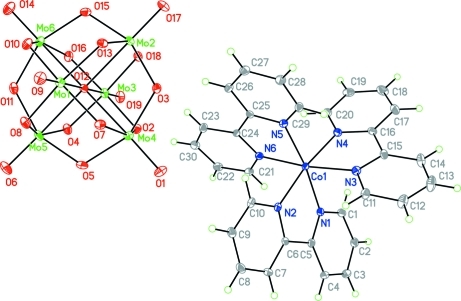
The molecular structure of (I) shoiwng displacement ellipsoids drawn at the 30% probability level; H atoms are given as spheres of arbitrary radius.

## supplementary crystallographic information

### Comment

There has been extensive interest in heteropolyoxometalates, owing to their fascinating properties and great potential applications in many fields such as, catalysis, material science, medicine, and magnetochemistry (Pope *et al.*, 1991). The organic amines, such as 3-(2-pyridyl)pyrazole and pyrazine, are used to effectively modify heteropolyoxomolybdates under hydrothermal condictions (Zhang, Dou *et al.*, 2009; Zhang, Wei, Sun *et al.*, 2009). Here, we describe the synthesis and structural characterization of the title compound.

As shown in Figure 1, the title compound consists of two subunits, *viz.* of a complex [Co(C_10_H_8_N_2_)_3_]^2+^ cation, one typical Lindqvist isopolyanion [Mo_6_O_19_]^2-^ anion (Dahlstrom *et al.*, 1982). The Co^2+^ cation is surrounded in a distorted octahedral coordination by six N atoms from three chelating 2,2'-bipyridine ligands. The Co—N bond lengths are in the range of 2.075 (5)—2.100 (5) Å, respectively, compared to reported one (Li & Xu, 2009).

The [Mo_6_O_19_]^2-^ polyoxoanion, possessing well known Lindquist structure, is formed by six MoO_6_ octahedra connected with each other through edge-sharing oxygen atoms and thus exhibits approximate Oh symmetry. Three kinds of oxygen atoms exist in the cluster, that is, terminal Oa, double-bridging oxygen Ob, and central oxygen Oc. Therefore, Mo—O band lengths can be grouped into three sets: Mo—Oa 1.669 (5)—1.682 (5) Å; Mo—Ob 1.888 (4)—1.951 (5) Å; and Mo—Oc 2.299 (4)—2.318 (4) Å; these bond distances have a rule of Mo—Oa<Mo—Ob<Mo—Oc. Comparing Mo=O bond distances with that of Lindqvist isopolyanion salt (Dahlstrom, 1982), Mo=O distances have no obvious change.

### Experimental

A mixture of 2,2'-bipyridine (0.5 mmoL, 0.07 g), molybdenum(VI) oxide (1 mmol, 0.14 g), oxalic aicd (10 mmol, 0.09), *p*-carboxyphenylboronic acid (0.3 mmoL, 0.05 g), and cobalt(II) sulfate heptahydrate (0.2 mmol, 0.05 g) in 14 ml distilled water was sealed in a 25 ml Teflon-lined stainless steel autoclave and was kept at 433 K for three days. Upon cooling, red blocks of (I) were obtained. Anal. Calc. for C_30_H_24_CoMo_6_N_6_O_19_: C, 25.58; H, 1.71; N, 5.97. Found: C, 22.38; H, 1.52; N, 5.78%.

### Refinement

All hydrogen atoms bound to carbon were refined using a riding model with distance C—H = 0.93 Å, *U*_iso_ = 1.2*U*_eq_ (C) for aromatic atoms.

### Crystal data

**Table:**

[Co(C_10_H_8_N_2_)_3_][Mo_6_O_19_]	*F*(000) = 2708
*M**_r_* = 1407.12	*D*_x_ = 2.376 Mg m^−^^3^
Monoclinic, *P*2_1_/*n*	Mo *K*α radiation, λ = 0.71073 Å
Hall symbol: -P 2yn	Cell parameters from 2413 reflections
*a* = 12.310 (2) Å	θ = 2.4–24.3°
*b* = 18.979 (4) Å	µ = 2.35 mm^−^^1^
*c* = 17.150 (4) Å	*T* = 296 K
β = 100.895 (3)°	Block, red
*V* = 3934.4 (14) Å^3^	0.12 × 0.10 × 0.08 mm
*Z* = 4	

### Data collection

**Table:**

Bruker APEXII CCD diffractometer	6500 independent reflections
Radiation source: fine-focus sealed tube	4649 reflections with *I* > 2σ(*I*)
graphite	*R*_int_ = 0.041
φ and ω scans	θ_max_ = 24.5°, θ_min_ = 2.2°
Absorption correction: multi-scan (*SADABS*; Bruker, 2001)	*h* = −14→13
*T*_min_ = 0.766, *T*_max_ = 0.834	*k* = −22→22
25652 measured reflections	*l* = −18→19

### Refinement

**Table:**

Refinement on *F*^2^	Primary atom site location: structure-invariant direct methods
Least-squares matrix: full	Secondary atom site location: difference Fourier map
*R*[*F*^2^ > 2σ(*F*^2^)] = 0.032	Hydrogen site location: inferred from neighbouring sites
*wR*(*F*^2^) = 0.092	H-atom parameters constrained
*S* = 1.00	*w* = 1/[σ^2^(*F*_o_^2^) + (0.041*P*)^2^ + 10.1791*P*] where *P* = (*F*_o_^2^ + 2*F*_c_^2^)/3
6500 reflections	(Δ/σ)_max_ = 0.003
559 parameters	Δρ_max_ = 0.65 e Å^−^^3^
0 restraints	Δρ_min_ = −0.54 e Å^−^^3^

### Special details

**Table:**

Geometry. All e.s.d.'s (except the e.s.d. in the dihedral angle between two l.s. planes) are estimated using the full covariance matrix. The cell e.s.d.'s are taken into account individually in the estimation of e.s.d.'s in distances, angles and torsion angles; correlations between e.s.d.'s in cell parameters are only used when they are defined by crystal symmetry. An approximate (isotropic) treatment of cell e.s.d.'s is used for estimating e.s.d.'s involving l.s. planes.
Refinement. Refinement of *F*^2^ against ALL reflections. The weighted *R*-factor *wR* and goodness of fit *S* are based on *F*^2^, conventional *R*-factors *R* are based on *F*, with *F* set to zero for negative *F*^2^. The threshold expression of *F*^2^ > σ(*F*^2^) is used only for calculating *R*-factors(gt) *etc*. and is not relevant to the choice of reflections for refinement. *R*-factors based on *F*^2^ are statistically about twice as large as those based on *F*, and *R*- factors based on ALL data will be even larger.

### Fractional atomic coordinates and isotropic or equivalent isotropic displacement parameters (Å^2^)

**Table:**

	*x*	*y*	*z*	*U*_iso_*/*U*_eq_	
C1	0.8841 (6)	0.1849 (4)	0.0079 (4)	0.0458 (17)	
H1	0.8962	0.1371	0.0008	0.055*	
C2	0.9125 (6)	0.2314 (4)	−0.0452 (4)	0.0523 (19)	
H2	0.9438	0.2154	−0.0873	0.063*	
C3	0.8947 (7)	0.3012 (4)	−0.0362 (4)	0.061 (2)	
H3	0.9130	0.3336	−0.0723	0.073*	
C4	0.8498 (6)	0.3235 (4)	0.0264 (4)	0.0526 (19)	
H4	0.8378	0.3712	0.0338	0.063*	
C5	0.8221 (5)	0.2735 (3)	0.0793 (4)	0.0395 (15)	
C6	0.7754 (5)	0.2926 (3)	0.1498 (4)	0.0355 (14)	
C7	0.7683 (6)	0.3610 (3)	0.1751 (4)	0.0478 (17)	
H7	0.7896	0.3982	0.1461	0.057*	
C8	0.7294 (6)	0.3736 (4)	0.2435 (4)	0.0554 (19)	
H8	0.7250	0.4196	0.2615	0.066*	
C9	0.6975 (5)	0.3189 (4)	0.2848 (4)	0.0472 (17)	
H9	0.6711	0.3265	0.3315	0.057*	
C10	0.7051 (5)	0.2527 (4)	0.2561 (4)	0.0425 (16)	
H10	0.6830	0.2152	0.2843	0.051*	
C11	0.5497 (5)	0.1943 (4)	0.0374 (4)	0.0478 (17)	
H11	0.5492	0.2282	0.0765	0.057*	
C12	0.4747 (6)	0.1997 (5)	−0.0318 (5)	0.063 (2)	
H12	0.4248	0.2369	−0.0398	0.076*	
C13	0.4737 (7)	0.1496 (5)	−0.0891 (5)	0.078 (3)	
H13	0.4225	0.1521	−0.1364	0.094*	
C14	0.5478 (6)	0.0964 (5)	−0.0764 (4)	0.065 (2)	
H14	0.5484	0.0622	−0.1151	0.078*	
C15	0.6226 (5)	0.0931 (4)	−0.0056 (4)	0.0427 (16)	
C16	0.7059 (6)	0.0373 (3)	0.0130 (4)	0.0447 (16)	
C17	0.7080 (7)	−0.0232 (4)	−0.0324 (5)	0.067 (2)	
H17	0.6542	−0.0295	−0.0779	0.080*	
C18	0.7867 (8)	−0.0727 (4)	−0.0117 (6)	0.079 (3)	
H18	0.7881	−0.1130	−0.0423	0.095*	
C19	0.8636 (7)	−0.0623 (4)	0.0551 (5)	0.063 (2)	
H19	0.9189	−0.0955	0.0709	0.076*	
C20	0.8592 (6)	−0.0022 (4)	0.0993 (4)	0.0542 (19)	
H20	0.9124	0.0043	0.1450	0.065*	
C21	1.0059 (5)	0.1421 (3)	0.2316 (4)	0.0452 (17)	
H21	1.0236	0.1587	0.1845	0.054*	
C22	1.0883 (6)	0.1384 (4)	0.2973 (4)	0.0529 (19)	
H22	1.1604	0.1515	0.2949	0.063*	
C23	0.9551 (5)	0.0950 (4)	0.3682 (4)	0.0472 (17)	
H23	0.9360	0.0791	0.4151	0.057*	
C24	0.8764 (5)	0.0987 (3)	0.2994 (3)	0.0329 (14)	
C25	0.7600 (5)	0.0778 (3)	0.2945 (4)	0.0330 (14)	
C26	0.7195 (6)	0.0507 (3)	0.3593 (4)	0.0454 (17)	
H26	0.7657	0.0443	0.4083	0.054*	
C27	0.6079 (6)	0.0339 (4)	0.3479 (4)	0.0519 (18)	
H27	0.5784	0.0159	0.3898	0.062*	
C28	0.5422 (6)	0.0433 (4)	0.2765 (4)	0.0511 (18)	
H28	0.4675	0.0316	0.2686	0.061*	
C29	0.5867 (5)	0.0701 (4)	0.2165 (4)	0.0451 (17)	
H29	0.5410	0.0771	0.1673	0.054*	
C30	1.0613 (6)	0.1148 (4)	0.3668 (5)	0.0531 (19)	
H30	1.1151	0.1124	0.4128	0.064*	
Co1	0.76522 (6)	0.13938 (4)	0.14146 (4)	0.02958 (19)	
Mo1	0.62552 (4)	0.16746 (3)	0.70535 (3)	0.03880 (16)	
Mo2	0.68058 (5)	0.03933 (3)	0.59227 (3)	0.03863 (15)	
Mo3	0.93267 (4)	0.09804 (3)	0.60537 (3)	0.04001 (16)	
Mo4	0.72000 (5)	0.20190 (3)	0.54351 (3)	0.04164 (16)	
Mo5	0.87672 (5)	0.22739 (3)	0.71735 (4)	0.04679 (17)	
Mo6	0.83628 (5)	0.06397 (3)	0.76648 (3)	0.04393 (17)	
N1	0.8397 (4)	0.2045 (3)	0.0694 (3)	0.0371 (12)	
N2	0.7428 (4)	0.2385 (3)	0.1891 (3)	0.0355 (12)	
N3	0.6238 (4)	0.1425 (3)	0.0512 (3)	0.0421 (13)	
N4	0.7816 (4)	0.0472 (3)	0.0790 (3)	0.0401 (13)	
N5	0.6935 (4)	0.0870 (3)	0.2251 (3)	0.0395 (13)	
N6	0.9018 (4)	0.1232 (3)	0.2313 (3)	0.0373 (12)	
O1	0.6778 (4)	0.2537 (3)	0.4646 (3)	0.0632 (14)	
O2	0.8518 (4)	0.1606 (2)	0.5229 (3)	0.0467 (11)	
O3	0.6467 (3)	0.1147 (2)	0.5161 (2)	0.0424 (11)	
O4	0.9808 (3)	0.1811 (2)	0.6620 (3)	0.0492 (12)	
O5	0.8119 (4)	0.2659 (2)	0.6171 (3)	0.0518 (12)	
O6	0.9511 (4)	0.2935 (3)	0.7643 (3)	0.0744 (17)	
O7	0.6093 (3)	0.2189 (2)	0.6099 (3)	0.0447 (11)	
O8	0.7392 (4)	0.2354 (2)	0.7496 (3)	0.0494 (12)	
O9	0.5168 (4)	0.1929 (3)	0.7438 (3)	0.0539 (12)	
O10	0.7046 (3)	0.1044 (2)	0.7871 (3)	0.0484 (12)	
O11	0.9111 (4)	0.1509 (3)	0.7929 (3)	0.0518 (12)	
O12	0.7786 (3)	0.13302 (19)	0.6550 (2)	0.0329 (9)	
O13	0.5755 (3)	0.0853 (2)	0.6474 (3)	0.0419 (11)	
O14	0.8815 (4)	0.0131 (3)	0.8463 (3)	0.0705 (16)	
O15	0.7458 (4)	0.0007 (2)	0.6930 (3)	0.0485 (12)	
O16	0.9500 (3)	0.0484 (2)	0.7010 (3)	0.0475 (12)	
O17	0.6077 (4)	−0.0283 (2)	0.5469 (3)	0.0600 (14)	
O18	0.8188 (3)	0.0303 (2)	0.5606 (2)	0.0438 (11)	
O19	1.0407 (4)	0.0735 (3)	0.5655 (3)	0.0565 (13)	

### Atomic displacement parameters (Å^2^)

**Table:**

	*U*^11^	*U*^22^	*U*^33^	*U*^12^	*U*^13^	*U*^23^
C1	0.057 (4)	0.042 (4)	0.041 (4)	−0.006 (3)	0.017 (3)	−0.004 (3)
C2	0.064 (5)	0.061 (5)	0.034 (4)	−0.010 (4)	0.015 (4)	−0.004 (4)
C3	0.086 (6)	0.055 (5)	0.043 (4)	−0.018 (4)	0.015 (4)	0.010 (4)
C4	0.079 (5)	0.036 (4)	0.041 (4)	−0.006 (4)	0.006 (4)	0.006 (3)
C5	0.036 (4)	0.048 (4)	0.032 (4)	−0.004 (3)	0.001 (3)	0.002 (3)
C6	0.036 (4)	0.033 (4)	0.034 (4)	−0.003 (3)	−0.002 (3)	−0.001 (3)
C7	0.061 (5)	0.030 (4)	0.050 (4)	−0.001 (3)	0.006 (4)	−0.002 (3)
C8	0.064 (5)	0.042 (4)	0.057 (5)	0.009 (4)	0.003 (4)	−0.016 (4)
C9	0.049 (4)	0.049 (4)	0.043 (4)	0.009 (3)	0.008 (3)	−0.011 (4)
C10	0.043 (4)	0.055 (4)	0.029 (4)	0.011 (3)	0.005 (3)	0.005 (3)
C11	0.048 (4)	0.055 (4)	0.040 (4)	0.011 (4)	0.009 (3)	−0.003 (3)
C12	0.043 (4)	0.089 (6)	0.056 (5)	0.028 (4)	0.007 (4)	0.004 (5)
C13	0.045 (5)	0.128 (8)	0.056 (5)	0.025 (5)	−0.001 (4)	−0.008 (6)
C14	0.053 (5)	0.091 (6)	0.048 (5)	0.005 (5)	0.002 (4)	−0.022 (4)
C15	0.036 (4)	0.054 (4)	0.038 (4)	−0.008 (3)	0.006 (3)	−0.006 (3)
C16	0.055 (4)	0.035 (4)	0.048 (4)	−0.009 (3)	0.017 (4)	−0.003 (3)
C17	0.083 (6)	0.051 (5)	0.060 (5)	−0.005 (4)	0.000 (4)	−0.018 (4)
C18	0.115 (8)	0.039 (5)	0.087 (7)	0.003 (5)	0.029 (6)	−0.021 (5)
C19	0.076 (6)	0.040 (4)	0.080 (6)	0.011 (4)	0.030 (5)	0.004 (4)
C20	0.055 (5)	0.050 (5)	0.057 (5)	0.012 (4)	0.011 (4)	0.006 (4)
C21	0.046 (4)	0.041 (4)	0.050 (4)	−0.004 (3)	0.013 (3)	0.005 (3)
C22	0.038 (4)	0.048 (4)	0.068 (5)	−0.006 (3)	0.000 (4)	0.006 (4)
C23	0.047 (4)	0.051 (4)	0.040 (4)	0.007 (3)	0.000 (3)	0.004 (3)
C24	0.045 (4)	0.025 (3)	0.029 (3)	0.005 (3)	0.008 (3)	−0.001 (3)
C25	0.039 (4)	0.026 (3)	0.034 (4)	−0.001 (3)	0.008 (3)	−0.005 (3)
C26	0.056 (5)	0.045 (4)	0.036 (4)	−0.001 (3)	0.011 (3)	0.003 (3)
C27	0.058 (5)	0.054 (4)	0.049 (4)	−0.008 (4)	0.022 (4)	0.004 (4)
C28	0.043 (4)	0.057 (5)	0.055 (5)	−0.011 (3)	0.013 (4)	0.003 (4)
C29	0.039 (4)	0.057 (4)	0.037 (4)	−0.010 (3)	0.003 (3)	0.004 (3)
C30	0.042 (4)	0.046 (4)	0.065 (5)	0.002 (3)	−0.006 (4)	0.005 (4)
Co1	0.0301 (4)	0.0305 (4)	0.0280 (4)	−0.0009 (3)	0.0050 (3)	0.0012 (3)
Mo1	0.0331 (3)	0.0401 (3)	0.0451 (3)	0.0020 (2)	0.0123 (3)	−0.0020 (3)
Mo2	0.0368 (3)	0.0331 (3)	0.0451 (4)	−0.0059 (2)	0.0056 (3)	−0.0064 (3)
Mo3	0.0320 (3)	0.0490 (4)	0.0402 (3)	0.0000 (3)	0.0099 (3)	−0.0069 (3)
Mo4	0.0430 (3)	0.0396 (3)	0.0428 (3)	0.0011 (3)	0.0094 (3)	0.0095 (3)
Mo5	0.0413 (3)	0.0481 (4)	0.0522 (4)	−0.0139 (3)	0.0117 (3)	−0.0186 (3)
Mo6	0.0384 (3)	0.0556 (4)	0.0369 (3)	0.0076 (3)	0.0049 (3)	0.0077 (3)
N1	0.042 (3)	0.039 (3)	0.030 (3)	−0.003 (2)	0.007 (2)	−0.002 (2)
N2	0.036 (3)	0.041 (3)	0.028 (3)	0.001 (2)	0.001 (2)	0.001 (2)
N3	0.037 (3)	0.045 (3)	0.045 (3)	0.001 (3)	0.008 (3)	0.003 (3)
N4	0.040 (3)	0.038 (3)	0.042 (3)	−0.001 (2)	0.007 (3)	0.000 (3)
N5	0.042 (3)	0.037 (3)	0.039 (3)	−0.004 (2)	0.006 (3)	0.004 (2)
N6	0.035 (3)	0.036 (3)	0.040 (3)	−0.005 (2)	0.005 (2)	0.003 (2)
O1	0.063 (3)	0.067 (3)	0.060 (3)	0.004 (3)	0.014 (3)	0.027 (3)
O2	0.046 (3)	0.053 (3)	0.044 (3)	−0.002 (2)	0.015 (2)	0.002 (2)
O3	0.035 (2)	0.049 (3)	0.039 (3)	−0.003 (2)	−0.004 (2)	0.001 (2)
O4	0.036 (3)	0.062 (3)	0.049 (3)	−0.012 (2)	0.007 (2)	−0.014 (2)
O5	0.054 (3)	0.032 (2)	0.073 (3)	−0.008 (2)	0.019 (3)	0.003 (2)
O6	0.063 (3)	0.069 (4)	0.090 (4)	−0.023 (3)	0.012 (3)	−0.036 (3)
O7	0.041 (3)	0.040 (3)	0.054 (3)	0.005 (2)	0.011 (2)	0.004 (2)
O8	0.044 (3)	0.051 (3)	0.056 (3)	−0.001 (2)	0.015 (2)	−0.021 (2)
O9	0.039 (3)	0.066 (3)	0.061 (3)	0.006 (2)	0.018 (2)	0.000 (3)
O10	0.043 (3)	0.064 (3)	0.042 (3)	0.007 (2)	0.016 (2)	0.009 (2)
O11	0.040 (3)	0.070 (3)	0.043 (3)	−0.004 (2)	0.003 (2)	−0.012 (2)
O12	0.032 (2)	0.031 (2)	0.038 (2)	−0.0016 (18)	0.0104 (18)	−0.0038 (19)
O13	0.034 (2)	0.038 (2)	0.054 (3)	−0.0053 (19)	0.008 (2)	0.001 (2)
O14	0.056 (3)	0.096 (4)	0.057 (3)	0.017 (3)	0.006 (3)	0.026 (3)
O15	0.050 (3)	0.036 (2)	0.059 (3)	−0.001 (2)	0.008 (2)	0.010 (2)
O16	0.039 (3)	0.059 (3)	0.043 (3)	0.007 (2)	0.005 (2)	−0.001 (2)
O17	0.057 (3)	0.043 (3)	0.079 (4)	−0.010 (2)	0.008 (3)	−0.018 (3)
O18	0.044 (3)	0.044 (3)	0.043 (3)	0.004 (2)	0.008 (2)	−0.010 (2)
O19	0.041 (3)	0.080 (4)	0.050 (3)	0.006 (2)	0.014 (2)	−0.009 (3)

### Geometric parameters (Å, °)

**Table:**

C1—N1	1.330 (8)	C25—C26	1.398 (8)
C1—C2	1.361 (9)	C26—C27	1.387 (9)
C1—H1	0.9300	C26—H26	0.9300
C2—C3	1.358 (10)	C27—C28	1.346 (9)
C2—H2	0.9300	C27—H27	0.9300
C3—C4	1.364 (10)	C28—C29	1.354 (9)
C3—H3	0.9300	C28—H28	0.9300
C4—C5	1.400 (9)	C29—N5	1.334 (8)
C4—H4	0.9300	C29—H29	0.9300
C5—N1	1.343 (8)	C30—H30	0.9300
C5—C6	1.479 (8)	Co1—N5	2.075 (5)
C6—N2	1.331 (7)	Co1—N6	2.078 (5)
C6—C7	1.376 (8)	Co1—N1	2.079 (5)
C7—C8	1.367 (9)	Co1—N4	2.081 (5)
C7—H7	0.9300	Co1—N2	2.091 (5)
C8—C9	1.358 (10)	Co1—N3	2.100 (5)
C8—H8	0.9300	Mo1—O9	1.671 (4)
C9—C10	1.359 (9)	Mo1—O7	1.884 (4)
C9—H9	0.9300	Mo1—O13	1.888 (4)
C10—N2	1.345 (7)	Mo1—O8	1.948 (4)
C10—H10	0.9300	Mo1—O10	1.957 (4)
C11—N3	1.331 (8)	Mo1—O12	2.310 (4)
C11—C12	1.362 (9)	Mo2—O17	1.671 (4)
C11—H11	0.9300	Mo2—O18	1.888 (4)
C12—C13	1.366 (11)	Mo2—O15	1.909 (4)
C12—H12	0.9300	Mo2—O3	1.929 (4)
C13—C14	1.350 (11)	Mo2—O13	1.948 (4)
C13—H13	0.9300	Mo2—O12	2.299 (4)
C14—C15	1.379 (9)	Mo3—O19	1.673 (4)
C14—H14	0.9300	Mo3—O16	1.868 (4)
C15—N3	1.350 (8)	Mo3—O4	1.888 (4)
C15—C16	1.467 (9)	Mo3—O18	1.950 (4)
C16—N4	1.336 (8)	Mo3—O2	1.968 (4)
C16—C17	1.390 (9)	Mo3—O12	2.318 (4)
C17—C18	1.349 (11)	Mo4—O1	1.673 (5)
C17—H17	0.9300	Mo4—O2	1.894 (4)
C18—C19	1.354 (11)	Mo4—O3	1.900 (4)
C18—H18	0.9300	Mo4—O5	1.951 (5)
C19—C20	1.377 (10)	Mo4—O7	1.961 (4)
C19—H19	0.9300	Mo4—O12	2.316 (4)
C20—N4	1.336 (8)	Mo5—O6	1.669 (5)
C20—H20	0.9300	Mo5—O8	1.884 (4)
C21—N6	1.330 (8)	Mo5—O5	1.900 (5)
C21—C22	1.368 (9)	Mo5—O11	1.939 (5)
C21—H21	0.9300	Mo5—O4	1.943 (4)
C22—C30	1.373 (10)	Mo5—O12	2.307 (4)
C22—H22	0.9300	Mo6—O14	1.682 (5)
C23—C30	1.365 (9)	Mo6—O10	1.887 (4)
C23—C24	1.379 (8)	Mo6—O11	1.902 (5)
C23—H23	0.9300	Mo6—O15	1.934 (4)
C24—N6	1.347 (7)	Mo6—O16	1.976 (4)
C24—C25	1.474 (8)	Mo6—O12	2.316 (4)
C25—N5	1.323 (7)		
			
N1—C1—C2	123.1 (7)	O17—Mo2—O18	103.3 (2)
N1—C1—H1	118.5	O17—Mo2—O15	102.9 (2)
C2—C1—H1	118.5	O18—Mo2—O15	88.73 (19)
C3—C2—C1	119.3 (7)	O17—Mo2—O3	103.1 (2)
C3—C2—H2	120.3	O18—Mo2—O3	87.96 (18)
C1—C2—H2	120.3	O15—Mo2—O3	153.89 (18)
C2—C3—C4	119.4 (7)	O17—Mo2—O13	102.9 (2)
C2—C3—H3	120.3	O18—Mo2—O13	153.82 (17)
C4—C3—H3	120.3	O15—Mo2—O13	86.36 (19)
C3—C4—C5	119.0 (7)	O3—Mo2—O13	85.28 (18)
C3—C4—H4	120.5	O17—Mo2—O12	179.2 (2)
C5—C4—H4	120.5	O18—Mo2—O12	77.50 (15)
N1—C5—C4	120.9 (6)	O15—Mo2—O12	77.28 (16)
N1—C5—C6	116.1 (5)	O3—Mo2—O12	76.72 (16)
C4—C5—C6	123.0 (6)	O13—Mo2—O12	76.34 (15)
N2—C6—C7	121.6 (6)	O19—Mo3—O16	104.5 (2)
N2—C6—C5	115.2 (5)	O19—Mo3—O4	104.3 (2)
C7—C6—C5	123.2 (6)	O16—Mo3—O4	89.9 (2)
C8—C7—C6	119.3 (7)	O19—Mo3—O18	102.9 (2)
C8—C7—H7	120.4	O16—Mo3—O18	88.10 (19)
C6—C7—H7	120.4	O4—Mo3—O18	152.37 (18)
C9—C8—C7	119.8 (6)	O19—Mo3—O2	102.0 (2)
C9—C8—H8	120.1	O16—Mo3—O2	153.38 (18)
C7—C8—H8	120.1	O4—Mo3—O2	86.13 (19)
C8—C9—C10	118.1 (6)	O18—Mo3—O2	83.50 (18)
C8—C9—H9	120.9	O19—Mo3—O12	177.44 (19)
C10—C9—H9	120.9	O16—Mo3—O12	77.75 (16)
N2—C10—C9	123.6 (6)	O4—Mo3—O12	76.77 (16)
N2—C10—H10	118.2	O18—Mo3—O12	75.87 (15)
C9—C10—H10	118.2	O2—Mo3—O12	75.71 (16)
N3—C11—C12	122.6 (7)	O1—Mo4—O2	103.8 (2)
N3—C11—H11	118.7	O1—Mo4—O3	104.5 (2)
C12—C11—H11	118.7	O2—Mo4—O3	88.71 (19)
C13—C12—C11	119.1 (7)	O1—Mo4—O5	102.2 (2)
C13—C12—H12	120.5	O2—Mo4—O5	88.06 (19)
C11—C12—H12	120.5	O3—Mo4—O5	153.15 (18)
C14—C13—C12	119.4 (7)	O1—Mo4—O7	103.3 (2)
C14—C13—H13	120.3	O2—Mo4—O7	152.92 (18)
C12—C13—H13	120.3	O3—Mo4—O7	86.39 (18)
C13—C14—C15	119.6 (7)	O5—Mo4—O7	84.46 (19)
C13—C14—H14	120.2	O1—Mo4—O12	178.4 (2)
C15—C14—H14	120.2	O2—Mo4—O12	77.12 (16)
N3—C15—C14	121.2 (7)	O3—Mo4—O12	76.83 (15)
N3—C15—C16	115.6 (6)	O5—Mo4—O12	76.46 (16)
C14—C15—C16	123.2 (6)	O7—Mo4—O12	75.83 (15)
N4—C16—C17	120.5 (7)	O6—Mo5—O8	103.8 (2)
N4—C16—C15	115.5 (6)	O6—Mo5—O5	104.2 (2)
C17—C16—C15	124.0 (7)	O8—Mo5—O5	89.3 (2)
C18—C17—C16	121.0 (8)	O6—Mo5—O11	102.1 (3)
C18—C17—H17	119.5	O8—Mo5—O11	87.6 (2)
C16—C17—H17	119.5	O5—Mo5—O11	153.44 (19)
C17—C18—C19	118.2 (8)	O6—Mo5—O4	102.9 (2)
C17—C18—H18	120.9	O8—Mo5—O4	153.17 (18)
C19—C18—H18	120.9	O5—Mo5—O4	86.3 (2)
C18—C19—C20	119.6 (8)	O11—Mo5—O4	84.69 (19)
C18—C19—H19	120.2	O6—Mo5—O12	177.9 (2)
C20—C19—H19	120.2	O8—Mo5—O12	77.15 (16)
N4—C20—C19	122.6 (7)	O5—Mo5—O12	77.63 (16)
N4—C20—H20	118.7	O11—Mo5—O12	75.97 (16)
C19—C20—H20	118.7	O4—Mo5—O12	76.05 (15)
N6—C21—C22	123.6 (6)	O14—Mo6—O10	104.1 (2)
N6—C21—H21	118.2	O14—Mo6—O11	103.5 (2)
C22—C21—H21	118.2	O10—Mo6—O11	89.9 (2)
C21—C22—C30	117.9 (7)	O14—Mo6—O15	103.6 (2)
C21—C22—H22	121.0	O10—Mo6—O15	88.0 (2)
C30—C22—H22	121.0	O11—Mo6—O15	152.55 (19)
C30—C23—C24	119.4 (7)	O14—Mo6—O16	102.5 (2)
C30—C23—H23	120.3	O10—Mo6—O16	153.36 (18)
C24—C23—H23	120.3	O11—Mo6—O16	84.39 (19)
N6—C24—C23	121.2 (6)	O15—Mo6—O16	85.39 (19)
N6—C24—C25	115.2 (5)	O14—Mo6—O12	178.3 (2)
C23—C24—C25	123.6 (6)	O10—Mo6—O12	77.53 (16)
N5—C25—C26	120.7 (6)	O11—Mo6—O12	76.43 (17)
N5—C25—C24	116.5 (5)	O15—Mo6—O12	76.39 (15)
C26—C25—C24	122.9 (6)	O16—Mo6—O12	75.83 (16)
C27—C26—C25	117.7 (6)	C1—N1—C5	118.3 (5)
C27—C26—H26	121.2	C1—N1—Co1	126.7 (4)
C25—C26—H26	121.2	C5—N1—Co1	113.9 (4)
C28—C27—C26	120.5 (6)	C6—N2—C10	117.6 (6)
C28—C27—H27	119.8	C6—N2—Co1	114.9 (4)
C26—C27—H27	119.8	C10—N2—Co1	127.3 (4)
C27—C28—C29	118.8 (7)	C11—N3—C15	118.0 (6)
C27—C28—H28	120.6	C11—N3—Co1	126.6 (5)
C29—C28—H28	120.6	C15—N3—Co1	114.0 (4)
N5—C29—C28	122.6 (6)	C20—N4—C16	118.1 (6)
N5—C29—H29	118.7	C20—N4—Co1	126.1 (5)
C28—C29—H29	118.7	C16—N4—Co1	115.7 (4)
C23—C30—C22	119.7 (7)	C25—N5—C29	119.8 (5)
C23—C30—H30	120.2	C25—N5—Co1	114.4 (4)
C22—C30—H30	120.2	C29—N5—Co1	125.3 (4)
N5—Co1—N6	78.9 (2)	C21—N6—C24	118.1 (6)
N5—Co1—N1	171.4 (2)	C21—N6—Co1	127.3 (4)
N6—Co1—N1	98.20 (19)	C24—N6—Co1	114.0 (4)
N5—Co1—N4	92.8 (2)	Mo4—O2—Mo3	116.7 (2)
N6—Co1—N4	96.5 (2)	Mo4—O3—Mo2	116.5 (2)
N1—Co1—N4	95.6 (2)	Mo3—O4—Mo5	117.2 (2)
N5—Co1—N2	93.2 (2)	Mo5—O5—Mo4	116.0 (2)
N6—Co1—N2	89.51 (19)	Mo1—O7—Mo4	116.6 (2)
N1—Co1—N2	78.62 (19)	Mo5—O8—Mo1	116.8 (2)
N4—Co1—N2	172.2 (2)	Mo6—O10—Mo1	116.3 (2)
N5—Co1—N3	97.0 (2)	Mo6—O11—Mo5	117.1 (2)
N6—Co1—N3	173.0 (2)	Mo2—O12—Mo5	179.7 (2)
N1—Co1—N3	86.7 (2)	Mo2—O12—Mo1	90.06 (13)
N4—Co1—N3	78.0 (2)	Mo5—O12—Mo1	89.99 (13)
N2—Co1—N3	96.3 (2)	Mo2—O12—Mo4	89.78 (14)
O9—Mo1—O7	103.4 (2)	Mo5—O12—Mo4	89.91 (13)
O9—Mo1—O13	103.9 (2)	Mo1—O12—Mo4	90.02 (13)
O7—Mo1—O13	90.01 (18)	Mo2—O12—Mo6	90.04 (13)
O9—Mo1—O8	103.0 (2)	Mo5—O12—Mo6	90.27 (14)
O7—Mo1—O8	86.9 (2)	Mo1—O12—Mo6	89.78 (13)
O13—Mo1—O8	152.92 (17)	Mo4—O12—Mo6	179.7 (2)
O9—Mo1—O10	102.8 (2)	Mo2—O12—Mo3	90.00 (13)
O7—Mo1—O10	153.66 (18)	Mo5—O12—Mo3	89.96 (13)
O13—Mo1—O10	86.59 (19)	Mo1—O12—Mo3	179.6 (2)
O8—Mo1—O10	84.4 (2)	Mo4—O12—Mo3	90.40 (13)
O9—Mo1—O12	178.6 (2)	Mo6—O12—Mo3	89.79 (13)
O7—Mo1—O12	77.40 (16)	Mo1—O13—Mo2	116.4 (2)
O13—Mo1—O12	77.16 (15)	Mo2—O15—Mo6	116.3 (2)
O8—Mo1—O12	75.91 (15)	Mo3—O16—Mo6	116.6 (2)
O10—Mo1—O12	76.38 (15)	Mo2—O18—Mo3	116.5 (2)

### Hydrogen-bond geometry (Å, °)

**Table:**

*D*—H···*A*	*D*—H	H···*A*	*D*···*A*	*D*—H···*A*
C2—H2···O11^i^	0.93	2.36	3.166 (9)	145
C4—H4···O17^ii^	0.93	2.52	3.165 (9)	127
C11—H11···O4^iii^	0.93	2.51	3.400 (8)	161
C12—H12···O2^iii^	0.93	2.47	3.277 (10)	145
C20—H20···O14^iv^	0.93	2.53	3.159 (9)	125
C22—H22···O8^v^	0.93	2.54	3.230 (9)	132
C26—H26···O18	0.93	2.58	3.459 (8)	157

Symmetry codes: (i) *x*, *y*, *z*−1; (ii) −*x*+3/2, *y*+1/2, −*z*+1/2; (iii) *x*−1/2, −*y*+1/2, *z*−1/2; (iv) −*x*+2, −*y*, −*z*+1; (v) *x*+1/2, −*y*+1/2, *z*−1/2.
